# News and Gossip

**Published:** 1868-08-15

**Authors:** 


### EDITORIAL.

### *News and Gossip.*

Among the recently announced deaths from sun-stroke, is that of W. T. G. Morton, the practical originator of anæsthesia for surgical purposes. Among those from chloroform is that of Gen. Charles G. Halpine ("*Miles O'Reilly*.") An absurd and cruel rumor is going round the press that the chloroform was taken with suicidal intent. We are assured, by reliable authority, that this statement is entirely unfounded. Meanwhile the practice of mitigating the suffering of neuralgia by inhaling this dangerous fluid, is becoming alarmingly frequent. Popular attention should be directed to its danger.——A correspondent wishes to know what kind of *Pessary* we prefer in our practice. We decline to answer—let each be fully persuaded in his own mind. In a tolerable experience of over twenty years, we have never recommended any kind, but what we regretted it.——The Board of Regents in Michigan have on their hands an infinitesimal elephant, and no place to put him. One Charles J. Hempel has been appointed Professor of the Principles and Practice of Homœopathy in the Medical Department of the University, but the late decision of the Supreme Court leaves him without a platform to spout upon, and only the poor privilege of signing the medical diplomas. What *will* they do with him?——The Trustees of the *Illinois Hospital for the Insane*, at Jacksonville, have issued a pamphlet which thoroughly demolishes the crazy agitators who have so earnestly attacked its management. The public notions on the subject of sanity and insanity are exceedingly vague, and evidently need "reconstruction." ——Chancellor Shackleford has decided that the appointment of Drs. Maddin, Callender, Nichol, Buchanan, Van S. Lindsey, and H. M. Compton to the chairs in the Medical College at Nashville "was unauthorized and void;" that Drs. Jennings, Eve, and Jones are entitled to fill the several chairs in the college to which they have been respectively appointed. ——Professors Bartholow and Blackman, of Cincinnati, are having a heated controversy.——The *Cincinnati Repertory* is out in favor of free medical education. We expect in its next number it will "go in" for free medical attendance upon the sick. Each of these would be excellent, provided the butchers, grocers, *et alii*, would act likewise.——The *British Medical Journal* recommends in suspension of confirmed opium eating, large doses of *Phosphoric Acid* and *Lupulin*. Subsequently *Zinc*, *Iron* and *Quinine*.——A "good article" of sparkling champagne is now made from petroleum. ——It is stated that the venerable N. D. Stebbins, M.D., of Detroit, is proposed by his friends for the chair of "Institutes of Medicine," in either the Ann Arbor or Detroit Colleges. It is urged that he is far in advance of the former, whilst he would act as a conservative force upon the Young America energy of the latter. From our knowledge of this excellently preserved specimen of the olden time, and our innate horror of the so-called Progress of these modern days, we cordially second his nomination. Liebig and Farraday; Claude Bernard and Virchow, Chambers and Flint, Agassiz, and all that *profanum vulgus* are "very tolerable and not to be endured." Let us go back to "first principles."——The Medical Department of Bowdoin College is to be removed from Brunswick to Portland.——Dr. Jacob Bigelow, of Boston, says that he has not been obliged to occupy his house or stay at home on account of any indisposition or malady whatever for the last half century. He attributes this exemption from disease to temperance, hard work, and ABSTINENCE FROM MEDICINE.

